# Human papilloma viruses

**DOI:** 10.1186/1742-4690-9-S1-I23

**Published:** 2012-05-25

**Authors:** Denise A Galloway

**Affiliations:** 1 Fred Hutchinson Cancer Research Center, Seattle, USA

## 

A group of approximately 13 human papillomaviruses (HPVs) are responsible for virtually all cervical cancers as well as the majority of vulvar, vaginal anal and penile cancers, as well as at least half of oropharyngeal cancers. Currently two prophylactic vaccines are highly efficacious in preventing HPV 16 and 18 associated disease, and the quadrivalent vaccine also prevents HPV 6 and 11 warts. Currently therapeutic vaccines targeting the viral E6 and E7 oncogenes are being developed. One topic that will be discussed is whether the prophylactic vaccine can have utility in any setting where individuals are already infected. Secondly, this review will discuss the basis of long-term immunity and whether it is afforded by both vaccination and natural infection. Finally, strategies to provide more broad based coverage to other HPV types will be discussed.

